# Advances in Food Quality Management Driven by Industry 4.0: A Systematic Review-Based Framework

**DOI:** 10.3390/foods14142429

**Published:** 2025-07-10

**Authors:** Fernanda Araujo Pimentel Peres, Beniamin Achilles Bondarczuk, Leonardo de Carvalho Gomes, Laurence de Castro Jardim, Ricardo Gonçalves de Faria Corrêa, Ismael Cristofer Baierle

**Affiliations:** School of Engineering, Federal University of Rio Grande, FURG, Santo Antônio da Patrulha CEP 95500-000, RS, Brazil; beniamin@furg.br (B.A.B.); leonardo.gomes@furg.br (L.d.C.G.); ldecastroj@furg.br (L.d.C.J.); ricardofariacorrea@furg.br (R.G.d.F.C.); ismaelbaierle@furg.br (I.C.B.)

**Keywords:** Quality 4.0, Industry 4.0, advanced manufacturing, food manufacturing, digital technologies

## Abstract

Integrating Industry 4.0 technologies into food manufacturing processes transforms traditional quality management practices. This study aims to understand how these technologies are applied across managerial quality functions in the food industry. A systematic literature review was conducted using the Scopus and Web of Science databases, selecting 69 peer-reviewed articles. The analysis identified quality control (QC) and quality assurance (QA) as the most frequently addressed functions. Sensor technology was the most cited, followed by blockchain and artificial intelligence, mainly supporting food safety, process monitoring, and traceability. In contrast, quality design (QD), quality improvement (QI), and quality policy and strategy (QPS) were underrepresented, revealing a gap in strategic and innovation-focused applications. Based on these insights, the Food Quality Management 4.0 (FQM 4.0) framework was developed, mapping the relationship between Industry 4.0 technologies and the five managerial quality functions, with food safety positioned as a transversal dimension. The framework contributes to academia and industry by offering a structured view of technological integration in food quality management and identifying future research and implementation directions. This study highlights the need for broader adoption of advanced technologies to improve transparency, responsiveness, and overall quality performance in the food sector.

## 1. Introduction

One of the major challenges in the food industry is ensuring product safety and quality, making food quality management (FQM) a critical activity [1,2]. Traditionally, quality assurance and control are central to quality systems in this sector [2,3]. However, a specific department should address quality systemically throughout the organization [3]. The realization of food quality is a complex system dependent on food and human behavior. For a profound analysis of this system, it is important to integrate theories from both technological and managerial disciplines. In this context, the food quality management model [4] was proposed as an integrated concept that addresses this holistic analysis.

This model structures quality management into five managerial functions—(i) quality design (QD), (ii) quality control (QC), (iii) quality improvement (QI), (iv) quality assurance (QA), and (v) quality policy and strategy (QPS)—highlighting the complexity of effectively managing food quality at all levels. Despite its conceptual clarity, the holistic implementation of these functions in the food industry remains limited. One barrier is the intensive use of data, analytical tools, and human resources required. Industry 4.0 technologies offer new opportunities to support these activities, becoming a growing field of interest. According to [5], the increase in automation and control in food processing is often driven by external factors such as new safety regulations that demand specific adaptations across food segments.

Moreover, food manufacturers are under increasing pressure to innovate, enhance performance, and adopt sustainable technologies [6]. While the COVID-19 pandemic accelerated digital transformation across the food supply chain, low technological readiness still hampers progress [6,7]. Compared to other sectors, the food industry lags in adopting Industry 4.0 solutions, remaining in the early stages of digitalization [8].

In this context, a literature review introduced the concept of Quality 4.0 in the food industry [9], focusing specifically on the automation and digitalization of food quality analyses to ensure speed, objectivity, and reliability—an approach aligned with the QC function of the food quality management model [4]. While Dias et al. [3] also conceptualized Quality 4.0, their analysis did not target a specific production sector. Two further reviews [6,10] explored Industry 4.0 technologies within the food industry; however, they did not elucidate the mechanisms by which these technologies support quality management. Drawing from this body of knowledge, Industry 4.0 technologies can be organized into four categories: (i) connectivity and integration, including cybersecurity, blockchain, and the Internet of Things (IoT); (ii) Cybernetics, covering digitalization and automation through smart sensors, robotics, artificial intelligence (AI), and machine learning (ML); (iii) data management, with big data and cloud technologies; (iv) simulation and extended reality, such as digital twins and cyber-physical systems. This article builds upon these contributions by expanding the scope beyond QC to encompass all five quality management functions. This represents a novel contribution, as it broadens the understanding of the subject by proposing an analysis of how activities related to quality management functions are being transformed through integration with Industry 4.0-enabling technologies.

Given the wide range of available technologies, there is an opportunity to understand how these solutions can support food quality management. However, there is still a lack of structured studies connecting specific Industry 4.0 technologies to the five managerial quality functions of food manufacturing. This gap limits the systemic understanding of how digital transformation can enhance quality management in this sector.

Therefore, through a systematic literature review, this study aims to analyze the state of the art regarding how Industry 4.0 technologies support the five managerial functions of food quality management in the food manufacturing process. The analysis of the review’s findings supports the proposed Food Quality Management 4.0 (FQM 4.0) framework. This framework evolves from a previously proposed framework in the literature [4] and illustrates which technologies are being employed to execute activities within each quality managerial function.

The first part of the article presents the quality managerial functions addressed in the selected studies and the Industry 4.0 technologies that have already been used or have the potential to be used to support these functions’ activities. Next, the FQM 4.0 framework is proposed. This paper does not aim to explore each implementation in depth but to provide an overview.

## 2. Materials and Methods

### 2.1. Study Design and Systematic Review Protocol

The study was motivated by the increasing importance of digital transformation in food production and the lack of integrative models linking Industry 4.0 technologies with food quality management practices. Based on this context, two research questions guided this study: (RQ1) Which quality managerial functions have been most explored in food manufacturing using Industry 4.0 technologies? (RQ2) How are digital technologies applied to support food quality management?

To conduct this systematic literature review, PRISMA (Preferred Reporting Items for Systematic Reviews and Meta-Analyses) guidelines were adopted [11].

Based on the analysis of the scientific literature, we identify the current state of knowledge and propose a conceptual framework that integrates technologies with quality functions in the food industry.

### 2.2. Search Strategy

The databases surveyed were Scopus and Web of Science. The choice was restricted to these two databases since they host all relevant JCR-indexed journals in the field of quality management. Keywords used in the search were as follows: ((“big data” OR “machine learning” OR “artificial intelligence” OR “cloud” OR “smart sensor*” OR “robotic*” OR “Internet of Things” OR “blockchain*” OR “cybersecurity” OR “digital twin*” OR “cyber-physical system*” OR “Industry 4.0” OR “advanced manufactur*” OR “automation*” OR “digitalization*” OR “digital transformation*” OR “intelligent manufactur*” OR “smart manufactur*” OR “smart factor*” OR “digital technolog*” OR “fourth industrial revolution”) AND (“food”) AND (“food quality” OR “food design” OR “food improvement” OR “food control” OR “food assurance” OR “food policy” OR “food strategy” OR “food quality management” OR “food management” OR “quality strategy” OR “quality policy” OR “quality control” OR “quality assurance” OR “quality design” OR “quality improvement”)), provided it was presented in the article’s title, abstract, or keywords. Boolean operators “AND” and “OR” combined word groups in the search. The search was carried out between on July 2023.

### 2.3. Study Selection

The articles were imported into Mendeley software v1.19.8 to remove duplicates. The inclusion criteria included (i) articles and reviews published in English in scientific journals and (ii) articles published since 2011, provided that this was the year in which the term “Industry 4.0” first appeared in a publication to describe the widespread integration of information and communication technology in industrial manufacturing [8]. The exclusion criteria were as follows: (i) articles that did not address any of the five quality managerial functions, (ii) articles that did not address quality in food manufacturing, (iii) articles that had to be paid to be read, (iv) articles that did not frequently mention Industry 4.0 technologies according to a predefined threshold.

Food manufacturing, as defined herein, encompasses only those stages executed by and impacting the operations of food industries, including but not limited to controlled-environment storage, selection, milling, drying, and packaging [12]. Consequently, the framework concentrates on the technological function of the techno-managerial model, defined as the “processing of food materials to food products” [4]. The scope excludes processes undertaken by other actors within the food system, which the Food and Agriculture Organization defines as food production, distribution, consumption, and disposal [13].

The selection process was independently conducted by six researchers who assessed the suitability of article titles and abstracts. The authors’ critical evaluation was based on the two questions presented in Section 2.1 to guide the exclusion of articles. The research team periodically convened during the selection process to resolve uncertainties and prevent discrepancies.

### 2.4. Data Extraction

Two independent authors (F.A.P.P. and I.C.B.) extracted and summarized the following data: (i) authors name, (ii) year of publication, (iii) location, (iv) article type, (v) food industry sector, (vi) quality managerial function addressed (based on the FQM model [4]), (vii) Industry 4.0 technologies discussed. Quantitative metrics (e.g., frequency of terms and technologies) were also used to support the qualitative insights. Microsoft Excel was used to compile the recorded data for additional analysis.

### 2.5. Data Synthesis and Data Analysis

A comprehensive review of the documents revealed numerous examples of activities linked to various quality functions supported by digital technologies. It was observed that explicit mentions of specific quality functions, such as quality design, quality control, quality improvement, quality assurance, and quality policy and strategy, were frequently absent. Consequently, to accurately categorize articles within each managerial quality function, we aligned the examples provided with the keywords or related terms listed in Table 1 [2,4].

For instance, if a document described developing a new quality process leveraging digital technology for its execution, the example was associated with the quality design managerial function. A pertinent illustration from [14] involved using digital technology to develop a new quality process for cooling horticultural products. Although “quality design” was not explicitly stated, this activity was attributed to that function due to its alignment with the established criteria. After initial categorization, the findings were reviewed by one author (F.A.P.P.) to ensure the accurate assignment of examples and prevent double-counting in the review’s indicators. Examples of digital technology use in supporting quality activities are detailed in Tables S1–S5 in the Supplementary Materials and will be further discussed in Section 3.2.

After categorizing the articles into managerial quality functions, we performed a qualitative content analysis technique using NVivo software (version 14). This analysis aimed to mine the most frequently mentioned Industry 4.0 technologies (Table 2) within each quality function. We used NVivo’s text search queries, including derived terms, and sorted the results in descending order, prioritizing articles with the highest frequency of technology mentions. To ensure the significance of our findings, we included articles for discussion in each managerial function only if they mentioned at least 1 of the 11 enabling technologies 50 times or more. This threshold was established based on the corpus’ size and characteristics, aligning with common practices in content analysis and text mining [6,15], where occurrences greater than 50 are considered significant. This rigorous frequency criterion ensured that only highly representative terms were included in the analysis, enhancing our findings’ consistency and robustness.

While presented as distinct categories in Table 2, these technologies frequently interrelate. For example, smart sensors (Cybernetics) often combine with the Internet of Things (IoT) (connectivity and integration) to facilitate data transfer across connected networks. Similarly, sensors (Cybernetics) collect large volumes of data that are then analyzed using AI and ML tools (Cybernetics) for big data analytics (data management).

### 2.6. Framework Development and Validation

Building on the insights from our systematic review, we developed the Food Quality Management 4.0 (FQM 4.0) framework. This framework conceptually links quality managerial functions with the application of digital technologies. It synthesizes the current scientific knowledge, highlights technology–function relationships, and pinpoints gaps and opportunities for future research and industrial practice. To validate the framework, we surveyed with 30 food industries in Brazil.

## 3. Results

### 3.1. Search and Characteristics of Studies

**Figure 1:**
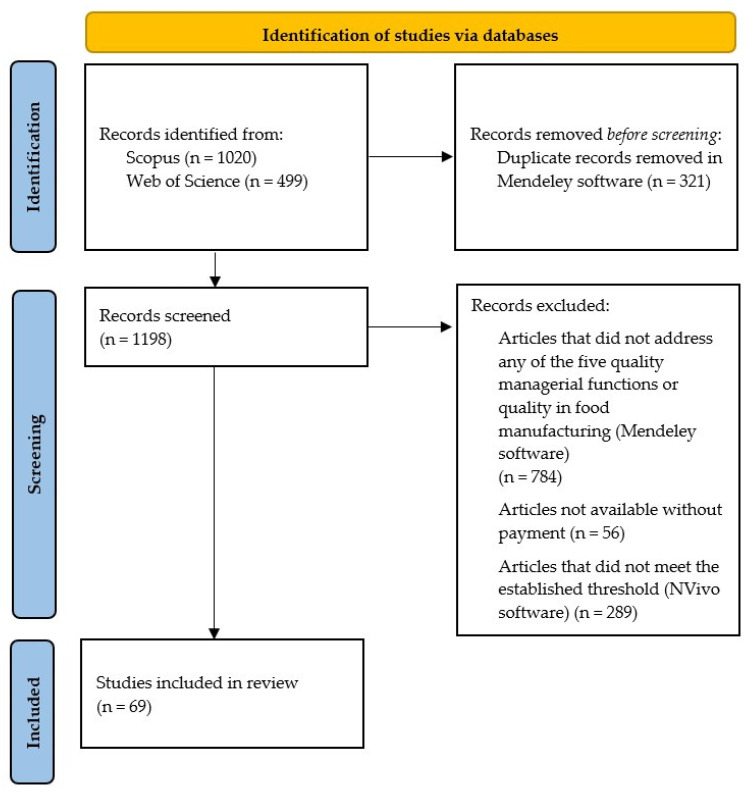
PRISMA flowchart illustrating the study selection process.

A total of 1519 articles were initially identified across the databases, and 321 duplicates were removed. After applying the exclusion criteria, 784 articles were excluded for not addressing any of the five quality managerial functions or not focusing on quality in food manufacturing. Additionally, 56 articles were excluded due to not being freely available, and 289 articles were eliminated because their mention of digital technologies did not meet the established threshold. Ultimately, 69 articles were synthesized and analyzed in this review (Figure 1).

The analysis of the 69 articles selected showed that 55% are literature reviews, and 80% have been published since 2020, indicating a growing interest in using 4.0 technologies to support food quality activities. The high number of systematic reviews may suggest the nascent maturity of food industries in practically implementing digital technologies for quality-related tasks. This aligns with the observation that new propositions (45% of the documents) are predominantly theoretical or at a laboratory scale, a point that will be further explored in Section 4.1.

An analysis of the most researched food industry sectors and countries with the most publications revealed that the Asian continent stands out, with China leading with 14 publications and India with 8. The sectors most frequently highlighted for their use of digital technologies in quality activities within these studies were horticultural products (fruits, vegetables, and their derivatives) with eight publications; animal products (meat and fish) with five publications; and diverse sectors such as tea, wheat flour, rice, spices, cocoa and chocolate, alcoholic beverages, cereals, and grains, cited in six publications. Other prominent countries include Canada, which has five publications, though only one specifically details the use of digital technologies in agricultural products [19]. Spain, the UK, and France each contributed three documents, with findings in the farming and meat sectors. These highly researched sectors indicate that developed countries are playing a leading role in this research area and that primary products, particularly those of animal and plant origin, are receiving the most attention.

### 3.2. Quality Managerial Functions Supported by Industry 4.0 Technologies

**Figure 2:**
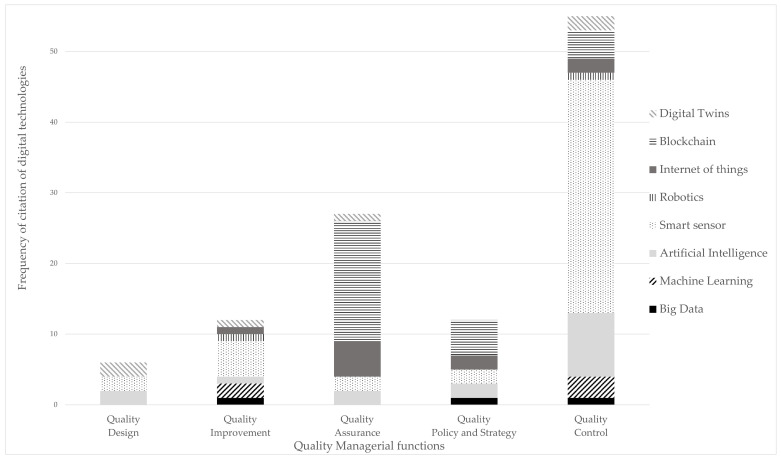
Frequency of citation of digital technologies divided by each managerial quality function.

Figure 2 illustrates the frequency of citation of digital technologies employed to support the activities within each managerial quality function. This figure surpasses the 69 articles cited in more than one enabling technology of Industry 4.0. This phenomenon may be attributed to the substantial number of literature reviews that presented illustrative applications of these technologies across various activities of food management. The “sensor” or “smart sensor” technology was the most frequently cited, notably mentioned in 39% of the examples. This prevalence is anticipated, considering this technology’s comparatively lower level of innovation and its frequent characterization as the gateway of Industry 4.0. Blockchain (23%), AI (14%), and IoT (9%) technologies were the second, third, and fourth most cited technologies, respectively. DT (5%), ML (4%), Big Data (3%), and Robotics (2%) received considerably fewer mentions. In the case of ML, this low incidence may be correlated with the observation that the term “machine learning” is less frequently cited than its constituent tools, such as k-NN, SVM, and ANN.

Cloud and cybersecurity technologies were not mentioned in any of the 69 articles. The lack of relevant mentions of these terms indicates that no research has been dedicated to these technologies that directly addresses quality in food manufacturing. The research focuses more on data collection and analysis than data storage or security. However, it is worth emphasizing the importance of these technologies for the food industry since a huge amount of data circulating on the web could lead to leaks of recipes or consumer data and would be detrimental to companies [6]. Cyber-physical systems technology was also not mentioned in the selected papers. Since DT, IoT, and robotics are not widespread like the others, cyber-physical systems, which combine these technologies, are still not being addressed in a representative way.

In regard to the managerial quality functions, QC was the most researched by the authors, with its activities being cited in 51% of the examples, followed by QA with 23% of the examples, QI with 11%, QPS with 10% of examples, and QD with only 4% of the examples. Based on the digital technologies categories [3], it can be said that QD and QI are mostly related to Cybernetics. QC is also related to Cybernetics, covering intelligent sensors and sensor technologies, robotics, AI, and ML. Additionally, this quality function presents examples of the connectivity and integration category. Finally, QA and QPS are quality managerial functions mostly associated with connectivity and integration, which address blockchain and IoT technologies.

#### 3.2.1. Quality Design

The application examples of digital technologies within quality design activities were categorized into three clusters (Table S1 in the Supplementary Materials): process development, product development, and new material development. Notably, no tools explicitly related to quality-oriented design, such as Quality Function Deployment (QFD), Failure Mode and Effects Analysis (FMEA), or Design of Experiments (DoE), were cited within the reviewed articles. Among the identified application examples supporting decision-making in QD are using sensors and smart sensors in conjunction with DT technology to develop novel quality processes [14] and simulations enabling enhanced responsiveness to customer quality requirements during product launch [28]. The application of AI was cited in support of pre-formulation studies [9], analyzing consumer information to support product development, and identifying materials capable of extending product shelf life [17]. This experiential learning, facilitated through simulated scenarios, prepares project stakeholders for real-world situations related to professional practice, enabling the development of interpersonal communication skills in project management within a safe, simulated environment [29]. It is pertinent to note that all the surveyed articles were of a review nature, with no novel methodologies proposed to enhance quality design activities.

#### 3.2.2. Quality Control

Activities related to quality control were identified in 51% of the articles selected. Eight technologies were mentioned to support quality control decision-making. The Cybernetics category was the most prominent since sensors, AI, and ML were most frequently mentioned, followed by the connectivity and integration category, covered by the application of blockchain and IoT technologies. This category’s activities were divided into nine clusters (Table S2 in the Supplementary Materials): classification, detection, monitoring, inspection, fraud prevention, prediction, assessment, new analysis method, and new sensor proposal.

Traditionally, food analysis has been determined using various destructive and time-consuming approaches with modest analytical performance. This highlights the urgent need to develop refined techniques connected to automated processes [9,30]. The relevant point is that many of the techniques and methods presented are potentially interesting in food manufacturing but are still only a reality at a laboratory scale [9].

Of the 46 articles allocated to quality control (QC), 50% reported activities supporting decision-making during product and process monitoring, and 46% cited detection activities, the most frequently mentioned clusters. Subsequently, activities related to fraud prevention (37%), assessment (35%), and classification (30%) were cited.

*Monitoring* entails the systematic tracking and collection of data over time. This process facilitates the measurement of performance against established targets and standards, as well as the identification of deviations [31]. The implementation of smart sensors for process monitoring was widely documented [32,33], with occasional instances of their integration alongside technologies such as IoT, AI, and blockchain. Illustrative examples encompass sensing to track and support decision-making concerning processes and their critical parameters (such as temperature, humidity, gas concentration, aroma, etc.) in the production of fresh produce, fruits, and vegetables [25,34,35] and in the monitoring of color in meats, the fatty acid composition of milk, and the determination of trans fatty acids in edible oils [23]. In another instance, a synergistic combination of blockchain and sensor technologies was developed for monitoring vital process parameters during the cold storage of frozen seafood [36] and for the provision of information regarding anomalous conditions of monitored food items to each authorized stakeholder within the supply chain, for corrective action implementation [37]. Integrating AI with other technologies is contingent upon the objectives of researchers and practitioners and the availability of pertinent data. To this end, a step-by-step procedure was proposed [18] to guide the integration process preceding the application of AI models for monitoring and inspection activities in the food industry.

Quality *detection* involves the identification of atypical behavior in comparison to normal operating data, indicating non-conformities [38]. Smart sensors [39,40,41,42], AI [43,44], and ML were the technologies most frequently employed in this activity. The integration of AI with sensors facilitates the detection of anomalies during the manufacturing process and enables the rectification of identified issues [16,23,45]. The application of e-noses has been extensively explored for the detection of contaminants in vegetable oils, grains, and spices [46], the adulteration of commodity foods [47], food-borne bacterial pathogens [16,48], toxins [15], and spoilage and fungal growth [45]. The detection of flaws during the fermentation of alcoholic beverages [49] was also noted. The principal attributes of e-noses include their economic viability, portability, ease of operation, non-invasive nature, and capacity for rapid analysis. Despite their increasing application, their widespread adoption in routine food industry practices remains limited [16,46,47].

*Fraud prevention* represents another significant category pertinent to quality control. Examples involving the utilization of smart sensors, AI, and blockchain were cited for classifying adulteration in powders and cereals [21], to rapidly detect subtle differences in aromatic profiles in coffee and pepper samples [50], and to ensure the transparency and authenticity of ingredients and the beer-making process [51]. The prevention of food adulteration, which predominantly occurs in high-value commodities, was a recurring theme in the articles in this section [19,52]. This constitutes an illicit practice characterized by intentionally adding physical substances to food products to reduce costs and augment sales profits. Related terms include “substitution,” which denotes adulteration with products of the same type, albeit not identical or of inferior quality, and “counterfeiting,” which refers to the sale of products that mimic genuine food items. Owing to the salience of this issue, there is an increasing demand for the development of precise and sensitive methodologies for identifying subtle and sophisticated adulteration in food products [21,44,47].

In quality control assessment, smart sensor technology was the most frequently cited. Specific examples involving the use of DT and blockchain were noted, such as the application of DT to support the verification of quality degradation in agricultural products stored in locations with limited accessibility for standard sensors [14], the integration of quality assessment smart contracts and models for the automatic quality assessment of fruit juice samples collected from different production stages [53], and the identification of lower quality shrimp products using blockchain technology [54].

Finally, smart sensors, AI, and ML technologies were employed within the classification category. This is exemplified by the application of deep learning for fruit classification [20], e-noses for the discrimination of different types of meat and fish samples [55], and tea sample flavors [56], ML methods for classifying bovine and ovine parenchymal organs [57] and identifying wheat classes [22], and the utilization of particle sensors combined with ML tools for food freshness classification [58].

The clusters of *inspection*, *prediction*, *new analysis*, and *new sensor development* [59,60,61,62] exhibited lower representation, and the application examples of digital technologies in the execution of these activities can be verified in Table S2 in the Supplementary Materials.

#### 3.2.3. Quality Improvement

Concerning quality improvement, the utilization of digital technologies to support activities related to this managerial function was exemplified in 10 articles, of which 50% cited the performance enhancement for sensors, 40% mentioned the continuous improvement of processes, 30% reported the reduction in process variability, and a mere 10% referred to waste reduction (Table S3 in the Supplementary Materials). Seven distinct technologies were mentioned in support of these activities.

The cluster of *performance enhancement for sensors* responsible for collecting data from food products and industrial processes presented examples involving the application of smart sensors and ML. Proposals addressing enhanced meat quality control aim to mitigate the impacts of sensor drift on smart sensors’ performance. Sensor drift occurs when gas sensors, such as e-noses, are adversely affected by other gases sharing common chemical properties and by environmental factors such as humidity, pressure, and temperature, impacting their accuracy and stability. ML techniques are implemented to compensate for these shortcomings [63,64,65]. Another challenge smart sensors face is low sensitivity and selectivity, or unsatisfactory levels of automation, in detecting ammonia, a compound indicative of food spoilage. To this end, gas sensor improvements have been proposed [66,67].

The cluster of *continuous improvement* reported the application of six digital technologies, including smart sensors and DT, to visualize the evolution of a process without the necessity of halting equipment or physically accessing the system to examine its state [28]; a smart IoT-based control system to provide reliable data from a modified cold storage room and issue necessary alerts in case of emergency based on real-time data analysis of post-harvest fruit quality [68]; and big data analysis by integrated systems to mitigate the potential for human error, thereby contributing to reduced costs and time and enhanced effectiveness and efficiency within the coffee supply [69]. The application of autonomous robots has been reported to provide skilled labor and reduce production costs in processes requiring material uniformity, such as packaging and palletizing. This is attributed to the heterogeneous formats of food products, which present challenges for their integration into more complex processes [6]. Furthermore, robotics has been implemented in food fractionation processes [70] to promote continuous improvement and a reduction in process variability.

The *process variability reduction* cluster explored the implementation of AI combined with big data to optimize batch blending processes and to consistently maintain food in optimum storage conditions [9]. Regarding the waste reduction category, DTs were mentioned for monitoring and predicting food processing stages, thereby contributing to optimizing the uniformity, performance, and sustainability of processes [28]. Only one article [9] mentioned the term “lean” concerning waste reduction. Notably, no article directly mentioned the implementation of a Six Sigma program as an example of process variability reduction.

#### 3.2.4. Quality Assurance

A total of 21 articles provided examples of quality assurance activities wherein decision-making was supported by five digital technologies (Table S4 in the Supplementary Materials). The QA activities were categorized into eight clusters: traceability (76%), quality audit (38%), quality system (24%), quality check (19%), quality management program (9%), safety hazards (5%), contract compliance (5%), and certification (5%).

Closely aligned with enhancing the *traceability* of the supply chain, wherein food manufacturing constitutes a critical link, blockchain and IoT technologies feature prominently in this section. Traceability within the food supply chain is the capacity to track and trace food, or substances intended for human or animal consumption, across all production, processing, and distribution stages. Blockchain technology has been applied to processes about traceability within the supply chain, contributing to more rapid, secure, and reliable data exchange among food supply chain participants, such as producers, transporters, and consumers. Furthermore, it facilitates regulatory compliance within industrial sectors efficiently and cost-effectively [26,71,72,73,74].

As one of its functional features, blockchain manages its transactions using smart contracts. By enforcing predefined terms and conditions, these contracts streamline operations, reduce costs, and help companies and regulatory authorities to promptly investigate and deal with potential safety hazards and avoid quality and safety incidents. These activities are often enhanced by IoT device integration and controlled user access [26,27,37,51,73,75].

Most application examples for blockchain and IoT in food traceability pertain to agricultural products and their entire journey from farm to fork, with food manufacturing considered a significant link in this chain, responsible for assessing the quality of incoming materials and ensuring delivery of quality products. The application of blockchain and IoT is cited to facilitate product recalls through data-driven decision-making [76]; compile an auditable history of any agricultural product, track all information online in real-time, readily identify contaminated products without necessitating the recall of an entire batch, and monitor the location and transportation conditions, verifying the characteristics of food products that utilize the cold chain [51,77]; minimize the production and distribution of unsafe or low-quality Xinzheng red jujube, thereby mitigating the potential for negative publicity, liability, and recalls [78]; issue a certificate for each food item verified at the point of purchase [72]; manage the cold chain for perishable products, such as the proposed ShrimpChain which will rank packaged shrimp according to the integrity and accuracy of authenticated data entered during various production stages [54]; enable consumers to readily access information concerning the extra virgin olive oil being purchased [10]; enhance agility in mitigating food crises by facilitating the precise detection and elimination of contamination sources [79]; and enable real-time monitoring and management of all communications and transactions within the agricultural supply chain, aiming to ensure food safety in a decentralized manner [80].

To support the execution of *quality audits*, blockchain technology has been applied to enable manufacturers to track, monitor, and audit the entire food process, including critical production stages [81], to achieve more streamlined and expedited auditing processes, reducing the need for extensive personnel involvement [73], and to manage the integration of routine production site audits within the framework of a Hazard Analysis and Critical Control Points (HACCP) system [79]. Furthermore, *quality systems* can be developed, as exemplified by the real-time information management and control of the rice supply chain based on a multi-chain collaboration architecture employing blockchain technology [82] and the application of smart contracts to establish incident alert levels for intelligent, real-time decision-making in the event of a food cold chain failure scenario for perishable products such as meat and fish [83]. The determination of foods’ origin with greater precision can be more readily facilitated through blockchain technology than traditional annual sampling within a third-party *certification* process [74].

Beyond blockchain, AI can be employed in *quality checks* to ensure, through facial and object recognition, that employees wear appropriate personal protective equipment, such as masks or caps, and that they verify the temperature and cleanliness of food [16].

Despite demonstrating significant potential for the agri-food supply chain, companies are still in the exploratory phase of utilizing blockchain, conducting proof-of-concept studies and tests for the future implementation of solutions [10,51]. Deficiencies in training and insufficient infrastructure investment impede the advancement of digital technologies implementation within agribusiness [26,84].

#### 3.2.5. Quality Policy and Strategy

Finally, a mere nine articles provided examples of applying five digital technologies to support decision-making concerning the formulation of food quality and safety policies and developing strategies (Table S5 in the Supplementary Materials). These quality policy and strategy activities were categorized into four distinct clusters: customer focus (56%), strategic analysis (44%), quality cost reduction (22%), and quality policy (11%).

Regarding *customer focus*, blockchain and IoT technologies were mentioned as enabling consumers to scrutinize product information and verify authenticity through the implementation of smart contracts, exemplified by the mitigation of fruit fraud risks [10], and as providing reliable and detailed product information on food origin, logistics details, and production and distribution processes to empower consumers to make informed and responsible purchasing decisions [76]. Extending beyond the food manufacturing process, the perceptions of 4017 tea consumers regarding blockchain-based traceability were assessed, and the resulting data was utilized to inform the strategic management of investments in food quality and safety programs [85].

In the conduction of *strategic analysis*, big data analysis proves advantageous for formulating and guiding an organization’s strategic direction [69]. A blockchain-based wireless sensor network monitoring system was cited to enhance quality control decision-making strategies [36]. Additionally, blockchain technology was mentioned to provide greater visibility of the taxes collected by the government and a more assertive allocation of resources that will support shrimp producers in meeting the demands of domestic and foreign markets and impel the export of seafood [54]. Examples of *quality cost reduction* and *quality policy* elaboration are detailed in Table S5, which is provided in the Supplementary Materials.

## 4. Discussion

### 4.1. Maturity of Quality Managerial Functions in Adoption of Digital Technologies

Analysis of the selected documents revealed the prevalence of the adoption of digital technologies for quality control (QC) and quality assurance (QA) activities, which aligns with previous findings [2,3]. This can be attributed to the fact that these functions are considered central to quality systems. Their primary focus is maintaining product and process parameters within specified technical tolerances and ensuring that consumers and customers meet their desired quality requirements. Since these functions are directly linked to customer needs concerning food standardization and safety, they are perceived by customers as adding the most value to companies.

In contrast, quality design (QD), quality improvement (QI), and quality policy and strategy (QPS) functions primarily concern internal company quality activities. These functions focus on enhancing product, process, and material development, identifying the root causes of problems to mitigate waste and reduce process tolerances, and defining long-term quality strategies and policies. Because these activities are less visible to customers, they may not be perceived as value-adding or worth a higher final product price [86]. This could explain why companies focus less on adopting digital technologies for these particular functions, reducing emphasis on these areas in academic research.

Additionally, the digital transformation of quality management functions faces several barriers [87]: technical (e.g., lack of standardized metrics), organizational (e.g., organizational resistance), and technological (e.g., insufficient technological infrastructure). These are all exacerbated by an economic barrier that demands investment in company diagnostics, employee training, and equipment acquisition. Therefore, it is logical that most propositions focus on activities that add more value in the eyes of consumers and customers, such as QC and QA.

New propositions are primarily related to quality control (QC) and quality assurance (QA) activities. An assessment of the technology readiness level (TRL) [88] of the presented propositions reveals a generally low maturity level (Table 3). Thirty percent of new propositions were classified at TRL 1–2, indicating that only the latest technological concept had been formulated. These technologies primarily addressed quality control (QC) activities, such as testing quality attributes and microbiological contamination of fruits and vegetables [34] and proposing a model to monitor food conditions and inform authorized users via sensors and blockchain technology [37]. Most new proposals, 54%, were at TRL 3–4, where a technology prototype had been developed and validated at the laboratory scale. Most of these cases are related to QC activities, including using sensors for classifying livestock internal organs [57], assessing *Staphylococcus aureus* exotoxin [62], and detecting subtle differences in the aromatic profiles of green coffee beans and Cayenne samples [50]. Propositions at higher TRLs were predominantly linked to quality assurance (QA) activities. Thirteen percent of proposals were between TRL 5 and 6, signifying validation in a relevant environment. An example is the development of a blockchain-based system for the quality traceability of Xinzheng red jujube, aimed at minimizing the production and distribution of unsafe or low-quality products, which was validated at Henan Xinzheng Xinxing Jujube Industry Corporation Ltd. (Xinzheng, China) [78]. Only one new proposition was identified in the TRL 7–8 range, where the system prototype is demonstrated in an operational environment. This example involved a prototype system for rice supply chain information management and control, built and applied to an enterprise in northeast China, where the model was analyzed and tested [82].

Just as the maturity level of new propositions is low, so is their current adoption by industries. Comparing QC and QA activities, it is evident that QA-related activities exhibit a higher maturity level (TRL ≥ 5). This suggests that proposals for quality assurance systems are more advanced and gain greater visibility from companies. Similarly, activities like traceability, audits, and certification can be incorporated into various businesses, whereas quality control analyses are often more tailored to specific food products.

No new model or framework propositions involved quality design (QD) activities. This presents an opportunity for quality engineering tools like Design of Experiments (DoE) and Quality Function Deployment (QFD) to have their stages supported by digital technologies.

### 4.2. Food Quality Management 4.0 Framework Proposition

**Figure 3:**
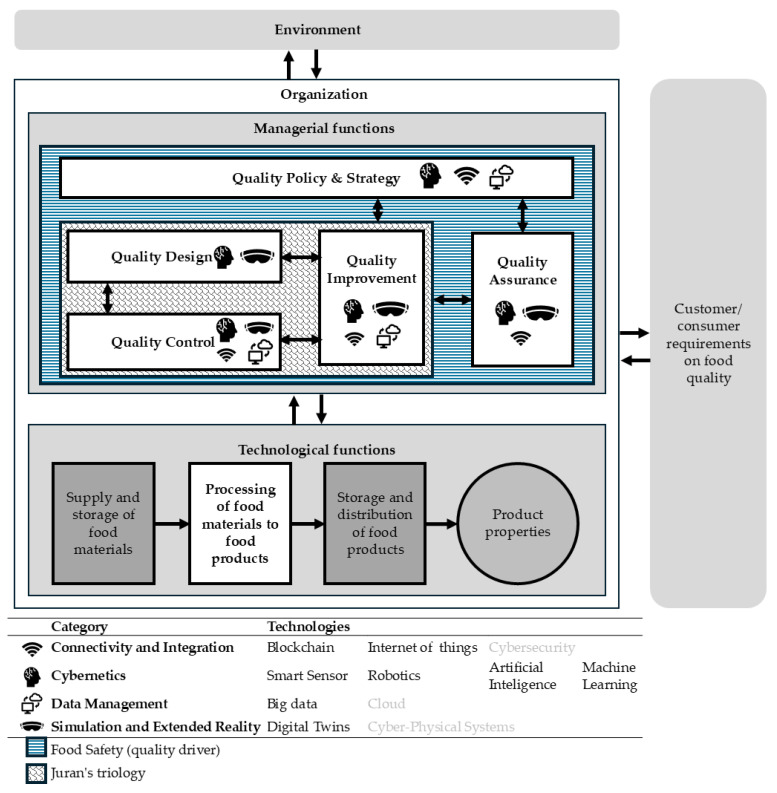
Food Quality Management 4.0 framework.

This section proposes the FQM 4.0 framework (Figure 3) as an evolution of the FQM model [4].

The framework’s consolidation is based on the most frequently mentioned Industry 4.0 technologies supporting quality management functions. This involved considering the citation frequency of digital technologies within each quality management function, as shown in Figure 2, and the four categories of Industry 4.0 technologies presented in Table 2. Consequently, a version of the FQM model [4] was enhanced to include the most prominent digital technologies for each management function. It is important to note that since this article filtered documents specifically addressing food manufacturing activities, the framework exclusively considers the technological function of “processing of food materials to food products,” highlighted in white within the framework.

The proposed framework highlights how digital technologies are gradually being integrated into food manufacturing, with a predominant focus on the Cybernetics category. Among these technologies, sensors are the most widely applied, supporting all five managerial quality functions—particularly QC and QI. Emerging technologies such as AI and ML are mainly associated with QC, enabling faster and more accurate evaluations.

Studies in the data management category have shown that big data supports QI by improving process efficiency and product quality. The simulation and extended reality category, represented by DT technologies, contributes to QD and QC by enabling virtual prototyping, predictive assessments, and real-time monitoring.

Connectivity and integration technologies, such as blockchain and IoT, are significantly associated with QA and QPS. Their integration enhances traceability, supports strategic decision-making, and increases transparency across the supply chain.

A major contribution of the framework is its emphasis on food safety as a transversal and critical component of food quality. Although not formally classified as a managerial quality function, food safety emerged as a central theme, mentioned in 94% of the analyzed articles and explicitly as “food safety” in 83%, especially in studies on sensors, blockchain, AI, and IoT. This underscores its relevance, driven by stricter regulations and the reputational risks of safety failures [89]. By incorporating food safety into the framework, we propose a more comprehensive understanding of quality in the food industry—where all managerial functions ultimately aim to ensure safe food production.

For practitioners, this framework provides a structured view of how Industry 4.0 technologies can be strategically aligned with quality functions to enhance food safety and performance. It offers a conceptual foundation for academia to explore the intersection between digital transformation and quality management in food manufacturing, identifying research gaps and guiding future empirical studies.

#### Practical Application: A Cocoa Manufacturing Illustrative Example

**Figure 4:**
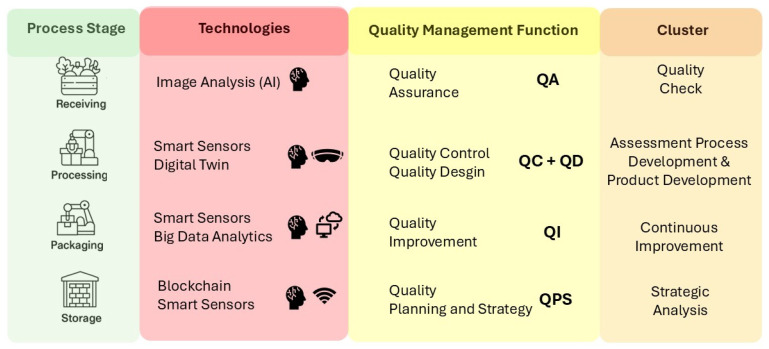
Illustrative application of the Food Quality Management 4.0 framework.

To demonstrate the practical applicability of the proposed framework, we developed a case study based on the cocoa manufacturing process (Figure 4). An example of the horticultural sector was chosen due to its prominence in empirical studies analyzed in our systematic literature review (SLR), highlighting its relevance in discussions concerning digital technologies and food quality management.

We divided the cocoa manufacturing process into four stages: receiving, processing, packaging, and storage. We identified relevant Industry 4.0 technologies from the literature for each stage and linked them to corresponding quality management functions. In the receiving stage, image analysis, powered by artificial intelligence (AI), detects foreign contaminants, such as stones and branches mixed with cocoa beans. This supports the quality assurance (QA) function (cluster: quality check). During processing, electronic noses determine the degree of roasting of cocoa beans. These smart sensors for this activity relate to the quality control (QC) function (cluster: assessment). Another quality activity incorporated at this stage is the definition of optimal process parameters for new products—for instance, a new cocoa origin—through simulation using digital twin (DT) technology. This eliminates the need for prototype testing and integrates quality design into the process (clusters: process development and product development).

At the packaging stage, sensors are integrated into the final product weighing process to collect extensive datasets. Big data analytics analyzes these to provide real-time insights for automatic process adjustments, reducing the frequency of human monitoring and increasing production efficiency. This promotes the quality improvement (QI) function (cluster: continuous improvement). Finally, a blockchain-based sensor network monitoring system in the storage stage supports strategic decision-making regarding stored products. This is achieved through mechanisms such as First-Expired-First-Out (FEFO) inventory management, dynamic expiry date implementation, and dynamic pricing systems, fostering increased transparency and trust and preventing data tampering. This activity contributes to the Quality Planning and Strategy (QPS) managerial function (cluster: strategic analysis).

### 4.3. FQM 4.0 Framework Validation in the Food Industry

A survey was administered to managers across 30 Brazilian food industries to validate the framework. Participants included quality managers (30%), quality coordinators (30%), quality analysts (13%), quality supervisors (10%), C-level executives (9%), quality directors (3%), and assistants (3%). The questionnaire featured both open and closed-ended questions concerning the use of digital technologies in their organizations’ quality activities and the drivers and barriers to adoption.

The industries represented a diverse range of sectors: 21% cocoa, chocolate, and confectionery manufacturing; 17% dairy products; 10% milling, starchy products, and animal feed; 10% spices, sauces, seasonings, and condiments; 10% alcoholic and non-alcoholic beverages; 7% preserved fruits, vegetables, and other plant-based products; 6% bakery products, biscuits, and crackers; 3% prepared foods and dishes; 3% vegetable and animal oils and fats; 3% slaughtering and meat product manufacturing; 10% other unclassified food product manufacturing.

The questionnaire provided examples of digital technology applications in quality processes, illustrating combinations of quality functions and Industry 4.0 categories as presented in the framework (Figure 3). Respondents could indicate all examples relevant to their company’s routines (Table 4).

Quality control (QC) activities supported by Cybernetics technologies were the most frequently reported. Common applications included using sensors for measuring temperature, humidity, pH changes, and the presence of gases (e.g., oxygen, ammonia, CO_2_). Computer vision for detecting product defects was also frequently mentioned.

Three technology categories were highlighted for quality assurance (QA) activities. IoT exemplified connectivity and integration for facilitating data transfer to support product traceability, and simulation and extended reality was exemplified by digital twins aiding hazard analysis and critical control points.

Consistent with the systematic review findings, companies cited quality improvement (QI) and quality design (QD) functions less frequently. QI was supported by Cybernetics (e.g., robots automating repetitive tasks and enhancing process standardization). QD was cited in conjunction with Cybernetics, involving artificial intelligence for developing product recipes aligned with customer preferences and machine-learning algorithms for analyzing customer data to inform new product development.

No company reported using digital technologies to support quality policy and strategy. Furthermore, 6% of the surveyed companies indicated they do not utilize any digital technologies in their quality processes.

Company representatives were asked to identify the primary drivers and barriers to adopting digital technologies in their quality routines. Key drivers included practicality in data collection, agility in correcting deviations, improved quality and reliability of records, accelerating decision-making, and reduced time for process control and routine quality activities. Conversely, the main barriers identified were the high implementation costs, lack of specialized labor, absence of an organizational culture that prioritizes investment in emerging technologies, and insufficient prioritization of the quality sector within companies, leading to investments primarily focused on increasing operational efficiency rather than quality activities.

The survey results align with the discussion on the technological maturity of the propositions in the literature. Despite consulting various company sectors and sizes, responses consistently pointed to using sensors for quality control as the primary application of digital technologies in quality routines. This suggests that low adoption rates might impact the overall technological readiness and diffusion of new technologies, corroborating the literature’s observation of a low readiness level for new quality methods, frameworks, and systems. The less disseminated these technologies are, the more expensive they become, leading to a more concentrated development in developed countries. The barriers identified by companies, such as high costs, personnel shortages, and organizational resistance, are also frequently reported in the academic literature [90]. To overcome these challenges, potential solutions include public–private partnerships for new technology development, pilot projects, or management training [91].

## 5. Conclusions

This study provides a systematic literature review to understand how digital technologies are being used to support the five managerial functions of food quality management in food manufacturing. Based on these findings, the Food Quality Management (FQM) 4.0 framework is proposed, offering a novel and integrative lens to understand the role of digital transformation in food quality management. The FQM 4.0 framework serves as a foundation for advancing academic research and supporting industrial practice in designing safer, smarter, and more resilient food production systems in the era of Industry 4.0. Theoretically, it organizes scattered research findings and provides a structured basis for future empirical studies. Practically, it assists companies in identifying technology gaps and guiding strategic investments in digital quality solutions.

This framework distinguishes itself from the existing literature by analyzing all quality functions that guide quality management in organizations, not just those corresponding to customer and consumer value streams, such as quality control (QC) and quality assurance (QA). A significant contribution of this work is addressing the existing gap in the evolution of processes related to quality improvement (QI), quality design (QD), and quality policy and strategy (QPS) activities in food manufacturing. A more holistic view of quality management in the food industry is a crucial exploration area. Furthermore, including an illustrative application case and the theoretical validation of the framework with the food industry enhance the robustness and clarity of the study’s propositions and discussions.

This research has certain limitations. First, the independent categorization of quality activity examples correlates each example with only one managerial quality function. In practice, however, a single activity may fulfill the requirements of multiple quality functions. Second, the prevalence of numerous literature reviews and conceptual or laboratory-scale method propositions diminishes practical guidance for companies interested in implementing these technologies in their routines. Finally, the conceptual nature of the framework limits its immediate adoption.

Furthermore, our findings lead to recommendations for future research. One area is the study of underexplored quality functions. A specific proposal involves examining the integration of technologies like sensors, big data analytics, and AI to support the collection and analysis of customer needs and the translation of these needs into product development requirements, thereby expanding studies on the quality design function.

Given the discussion on the low technological readiness level of the proposed methods in the literature, another emerging theme for future research is the necessity of real-world applications of the FQM 4.0 framework to validate the proposal. One suggestion is to explore the framework across different food industry sectors—such as fruits, vegetables, meat, fish, grains, and beverages—to understand which technologies are most suitable for each industrial branch. Lastly, while this framework primarily focused on quality activities performed in processing food materials into food products, one suggested extension is to analyze these activities within the scope of other technological functions such as the supply and storage of food materials and the storage and distribution of food products.

## Figures and Tables

**Table 1:** Aims and keywords related to each quality managerial function.

Quality Managerial Function	Aims	Keywords or Related Terms
Quality Design	Aims to incorporate quality into activities related to developing processes, products, or materials. These activities must be related to customers’ interests regarding a safer and higher quality product.	Process development Product development New material development Quality Function Deployment (QFD) Failure Mode and Effects Analysis (FMEA) Design of Experiments (DoE) Customer satisfaction Customer expectation Customer dissatisfaction
Quality Control	Aims to ensure that the variation in products and processes remains within a certain tolerance that is considered acceptable. Thus, compliance with specifications is assessed, and, where appropriate, interventions are made.	Statistical process control Acceptance sampling Visual inspection New analysis method New sensor proposal Inspection Classification Fraud Prediction Monitoring Assessment Detection
Quality Improvement	Aims to improve the quality system with a focus on causes and solutions through the change of people, processes, and resources to bring them to a higher level of quality, working with the reduction in tolerance in the production process.	Waste reduction Continuous improvement Lean Manufacturing Process variability reduction Six Sigma Lean sigma Metrics dashboards Performance enhancement sensor
Quality Assurance	Aims to control the quality system, its methods, and evaluations and to assure consumers and customers that the quality requirements have been met.	Quality management programs Hazard Analysis and Critical Control Points (HACCP) International Organization for Standardization (ISO) British Retail Consortium (BRC) International Featured Standards (IFS) Quality check Quality system Traceability Quality Audit Contract compliance Safety hazards Certification
Quality Policy and Strategy	Aims to define long-term food quality objectives and targets and how to achieve them through the quality system.	Total quality management Customer focus Strategic analysis Strategic partnership Food safety strategy Organizational culture Quality cost analysis Quality Strategy Development Quality policy

Source: [2,4].

**Table 2:** Enabling technologies of Industry 4.0.

Category	Industry 4.0 Technologies	Definition
Cybernetics	Smart Sensor	Sensors are transducers that measure physical and chemical quantities and convert them into electrical signals. They are the gateway to enabling Industry 4.0, ensuring better food quality and safety through low-cost, fast, reliable, and cost-effective detection methods [6,15]. Smart sensors can be classified into physical sensors, which measure temperature, humidity, and pressure in the food or vibration during transportation; chemical sensors that measure changes in pH and variations in gas concentrations (such as oxygen and carbon dioxide); and biological sensors that mimic the senses of the human body such as smell, sight, and taste [6,15,16].
Cybernetics	Artificial Intelligence or AI	AI technology is often associated with sensors. AI involves the development of algorithms and computer models that allow machines to process and analyze a large volume of data, identify patterns and relationships, and make predictions or decisions based on these analyses [17]. This technology is thus said to simulate human thinking and intelligence, learning ability, and knowledge storage [10,18,19], allowing answers to complex questions to be discovered [20].
Cybernetics	Machine Learning or ML	ML is a subcategory of AI [18,20,21,22]. This technology relates to developing and applying algorithms that can learn the patterns present in data and convert empirical data, using it to make classifications and predictions [21,22,23]. Examples of ML algorithms include artificial neural network (ANN), k-nearest neighbor (k-NN), support vector machine (SVM), decision trees (DTr), random forest (RF), and genetic algorithms. Deep learning is a subdivision of ML used for pattern recognition and decision-making [6,18,20].
Cybernetics	Robotics	Robotics is considered another sub-area of AI [20]. Autonomous robots have been reported to provide skilled labor and reduce production costs [6].
Data Management	Big Data	Decision-making based on analyzing a massive amount of data generated by operations, which undergo the digitization and automation of their processes, is related to big data technology [6,24].
Data Management	Cloud	Cloud is understood as a digital infrastructure used to store large amounts of data generated, whether personal or corporate [6].
Connectivity and Integration	Internet of Things or IoT	IoT, considered an essential dimension of Industry 4.0 [3], allows humans, objects, and things to connect and communicate at any time and anywhere. IoT systems consist of a network of physical objects with embedded technology to detect, communicate, and interact with their internal states or the external environment [25]. In the manufacturing environment, it enables data transfer between interconnected computer devices and industrial machinery [6].
Connectivity and Integration	Blockchain	Blockchain is an inviolable, transparent, decentralized, and, therefore, reliable technology that stores each environ transaction using cryptographic hashes [26,27].
Connectivity and Integration	Cybersecurity	Cybersecurity is the process and technology that support protecting information and technology systems [6].
Simulation and Extended Reality	Digital Twins or DT	DT technology is a virtual product, process, or device representation. A twin connects to the real world via sensors and provides real-time data to the virtual twin [6,14,28].
Simulation and Extended Reality	Cyber-physical systems	Cyber-physical systems have a strong relationship with DT, IoT, and robotics, as they integrates the physical and virtual worlds [6].

**Table 3:** The technology readiness level (TRL) of the proposals.

TRL	% Proposals	Quality Function Most Mentioned
1–2	30	QC (48%)
3–4	54	QC (63%)
5–6	13	QA (75%
7–8	3	QA (100%)
9	0	

**Table 4:** Managerial quality functions and Industry 4.0 technology adoption in food industries.

Managerial Quality Functions Supported by Industry 4.0 Category	%
QC and Cybernetics	72
QI and Cybernetics	6
QA and Connectivity and Integration	5
QA and Simulation and Extended Reality	3
QD and Cybernetics	3
QI and Data Management	3
QA and Cybernetics	1
Other	1
None	6
Total	100%

## Data Availability

The original contributions presented in this study are included in the article/Supplementary Materials. Further inquiries can be directed to the corresponding author.

## Supplementary Materials

The following supporting information can be downloaded at https://www.mdpi.com/article/10.3390/foods14142429/s1, Table S1. Quality design activities supported by digital technologies; Table S2. Quality control activities supported by digital technologies; Table S3. Quality improvement activities supported by digital technologies; Table S4. Quality assurance activities supported by digital technologies; Table S5. Quality policy and strategy activities supported by digital technologies.

## Abbreviations

The following abbreviations are used in this manuscript:

FQM	Food Quality Management
QD	Quality Design
QC	Quality Control
QI	Quality Improvement
QA	Quality Assurance
QPS	Quality Policy and Strategy
AI	Artificial Intelligence
ML	Machine Learning
IoT	Internet of Things
BD	Big Data Analysis
DT	Digital Twin
QFD	Quality Function Deployment
DoE	Design of Experiments
HACCP	Hazard Analysis and Critical Control Points

## References

[1] Lim S.A.H., Antony J. (2016). Statistical Process Control Readiness in the Food Industry: Development of a Self-Assessment Tool. Trends Food Sci. Technol..

[2] Dora M., Kumar M., Van Goubergen D., Molnar A., Gellynck X. (2013). Food Quality Management System: Reviewing Assessment Strategies and a Feasibility Study for European Food Small and Medium-Sized Enterprises. Food Control.

[3] Dias A.M., Carvalho A.M., Sampaio P. (2022). Quality 4.0: Literature Review Analysis, Definition and Impacts of the Digital Transformation Process on Quality. Int. J. Qual. Reliab. Manag..

[4] Luning P.A., Marcelis W.J. (2007). A Conceptual Model of Food Quality Management Functions Based on a Techno-Managerial Approach. Trends Food Sci. Technol..

[5] Kondakci T., Zhou W. (2017). Recent Applications of Advanced Control Techniques in Food Industry. Food Bioprocess Tech..

[6] Hassoun A., Aït-Kaddour A., Abu-Mahfouz A.M., Rathod N.B., Bader F., Barba F.J., Biancolillo A., Cropotova J., Galanakis C.M., Jambrak A.R. (2023). The Fourth Industrial Revolution in the Food Industry—Part I: Industry 4.0 Technologies. Crit. Rev. Food Sci. Nutr..

[7] Ilyukhin S.V., Haley T.A., Singh R.K. (2001). A Survey of Control System Validation Practices in the Food Industry. Food Control.

[8] Schuh G., Anderl R., Gausemeier J., ten Hompel M., Wahlster W. (2020). Industrie 4.0 Maturity Index: Managing the Digital Transformation of Companies.

[9] Hassoun A., Jagtap S., Garcia-Garcia G., Trollman H., Pateiro M., Lorenzo J.M., Trif M., Rusu A.V., Aadil R.M., Šimat V. (2023). Food Quality 4.0: From Traditional Approaches to Digitalized Automated Analysis. J. Food Eng..

[10] Hassoun A., Kamiloglu S., Garcia-Garcia G., Parra-López C., Trollman H., Jagtap S., Aadil R.M., Esatbeyoglu T. (2023). Implementation of Relevant Fourth Industrial Revolution Innovations across the Supply Chain of Fruits and Vegetables: A Short Update on Traceability 4.0. Food Chem..

[11] Page M.J., McKenzie J.E., Bossuyt P.M., Boutron I., Hoffmann T.C., Mulrow C.D., Shamseer L., Tetzlaff J.M., Akl E.A., Brennan S.E. (2021). The PRISMA 2020 Statement: An Updated Guideline for Reporting Systematic Reviews. BMJ.

[12] Guruswamy S., Pojić M., Subramanian J., Mastilović J., Sarang S., Subbanagounder A., Stojanović G., Jeoti V. (2022). Toward Better Food Security Using Concepts from Industry 5.0. Sensors.

[13] (2018). FAO Sustainable Food Systems—Concept and Framework. Sustainable Food Systems—Concept and Framework.

[14] Defraeye T., Shrivastava C., Berry T., Verboven P., Onwude D., Schudel S., Bühlmann A., Cronje P., Rossi R.M. (2021). Digital Twins Are Coming: Will We Need Them in Supply Chains of Fresh Horticultural Produce?. Trends Food Sci. Technol..

[15] Alahi M.E.E., Mukhopadhyay S.C. (2017). Detection Methodologies for Pathogen and Toxins: A Review. Sensors.

[16] Hassoun A., Jagtap S., Trollman H., Garcia-Garcia G., Abdullah N.A., Goksen G., Bader F., Ozogul F., Barba F.J., Cropotova J. (2023). Food Processing 4.0: Current and Future Developments Spurred by the Fourth Industrial Revolution. Food Control.

[17] Taneja A., Nair G., Joshi M., Sharma S., Sharma S., Jambrak A.R., Roselló-Soto E., Barba F.J., Castagnini J.M., Leksawasdi N. (2023). Artificial Intelligence: Implications for the Agri-Food Sector. Agronomy.

[18] Mavani N.R., Ali J.M., Othman S., Hussain M.A., Hashim H., Rahman N.A. (2022). Application of Artificial Intelligence in Food Industry—A Guideline. Food Eng. Rev..

[19] Misra N.N., Dixit Y., Al-Mallahi A., Bhullar M.S., Upadhyay R., Martynenko A. (2022). IoT, Big Data, and Artificial Intelligence in Agriculture and Food Industry. IEEE Internet Things J..

[20] Addanki M., Patra P., Kandra P. (2022). Recent Advances and Applications of Artificial Intelligence and Related Technologies in the Food Industry. Appl. Food Res..

[21] Kang Z., Zhao Y., Chen L., Guo Y., Mu Q., Wang S. (2022). Advances in Machine Learning and Hyperspectral Imaging in the Food Supply Chain. Food Eng. Rev..

[22] Saha D., Manickavasagan A. (2021). Machine Learning Techniques for Analysis of Hyperspectral Images to Determine Quality of Food Products: A Review. Curr. Res. Food Sci..

[23] Ropodi A.I., Panagou E.Z., Nychas G.-J.E. (2016). Data Mining Derived from Food Analyses Using Non-Invasive/Non-Destructive Analytical Techniques; Determination of Food Authenticity, Quality & Safety in Tandem with Computer Science Disciplines. Trends Food Sci. Technol..

[24] Ishwarappa, Anuradha J. (2015). A Brief Introduction on Big Data 5Vs Characteristics and Hadoop Technology. Procedia Comput. Sci..

[25] Onwude D.I., Chen G., Eke-Emezie N., Kabutey A., Khaled A.Y., Sturm B. (2020). Recent Advances in Reducing Food Losses in the Supply Chain of Fresh Agricultural Produce. Processes.

[26] Feng H.H., Wang X., Duan Y.Q., Zhang J., Zhang X.S. (2020). Applying Blockchain Technology to Improve Agri-Food Traceability: A Review of Development Methods, Benefits and Challenges. J. Clean. Prod..

[27] Tanwar S., Parmar A., Kumari A., Jadav N.K., Hong W.C., Sharma R. (2022). Blockchain Adoption to Secure the Food Industry: Opportunities and Challenges. Sustainability.

[28] Henrichs E., Noack T., Piedrahita A.M.P., Salem M.A., Stolz J., Krupitzer C. (2022). Can a Byte Improve Our Bite? An Analysis of Digital Twins in the Food Industry. Sensors.

[29] Tinoco E., Lima R.M., Mesquita D., Souza M.C. (2023). Using Scenarios for the Development of Personal Communication Competence in Project Management. Int. J. Proj. Organ. Manag..

[30] Matindoust S., Baghaei-Nejad M., Abadi M.H.S., Zou Z., Zheng L.R. (2016). Food Quality and Safety Monitoring Using Gas Sensor Array in Intelligent Packaging. Sens. Rev..

[31] Peres F.A.P., Peres T.N., Fogliatto F.S., Anzanello M.J. (2020). Strategies for Synchronizing Chocolate Conching Batch Process Data Using Dynamic Time Warping. J. Food Sci. Technol..

[32] Ahmadihaji A., Izquierdo R., Shih A. (2023). From Chip-Based to Chipless RFID Sensors: A Review. IEEE Sens. J..

[33] Kim C., Lee K.K., Kang M.S., Shin D.M., Oh J.W., Lee C.S., Han D.W. (2022). Artificial Olfactory Sensor Technology That Mimics the Olfactory Mechanism: A Comprehensive Review. Biomater. Res..

[34] Bandal A., Thirugnanam M. (2016). Quality Measurements of Fruits and Vegetables Using Sensor Network. Proceedings of the 3rd International Symposium on Big Data and Cloud Computing, ISBCC 2016.

[35] Saravanan P., Sathish Kumar S. (2013). Sensor Grid Middleware Architecture for Food Quality Control Units. Res. J. Pharm. Biol. Chem. Sci..

[36] Feng H., Wang W., Chen B., Zhang X. (2020). Evaluation on Frozen Shellfish Quality by Blockchain Based Multi-Sensors Monitoring and SVM Algorithm during Cold Storage. IEEE Access.

[37] Hameed H., Zafar N.A., Alkhammash E.H., Hadjouni M. (2022). Blockchain-Based Formal Model for Food Supply Chain Management System Using VDM-SL. Sustainability.

[38] Peres F.A.P., Peres T.N., Fogliatto F.S., Anzanello M.J. (2019). Fault Detection in Batch Processes through Variable Selection Integrated to Multiway Principal Component Analysis. J. Process Control.

[39] Reig C., Avila-Navarro E. (2014). Printed Antennas for Sensor Applications: A Review. IEEE Sens. J..

[40] Zhu P.H., Wang Y.C., Ma P., Li S.S., Fan F.Q., Cui K., Ge S.G., Zhang Y., Yu J.H. (2019). Low-Power and High-Performance Trimethylamine Gas Sensor Based on n-n Heterojunction Microbelts of Perylene Diimide/CdS. Anal. Chem..

[41] Anisimov D.S., Abramov A.A., Gaidarzhi V.P., Kaplun D.S., Agina E.V., Ponomarenko S.A. (2023). Food Freshness Measurements and Product Distinguishing by a Portable Electronic Nose Based on Organic Field-Effect Transistors. ACS Omega.

[42] Abbatangelo M., Núñez-Carmona E., Sberveglieri V. (2019). Novel Equipment for Food Quality Control: An IoT Nanowire Gas Sensors Array. Chem. Eng. Trans..

[43] Wang D.Y., Zhang M., Mujumdar A.S., Yu D.X. (2022). Advanced Detection Techniques Using Artificial Intelligence in Processing of Berries. Food Eng. Rev..

[44] Othman S., Mavani N.R., Hussain M.A., Abd Rahman N., Ali J.M. (2023). Artificial Intelligence-Based Techniques for Adulteration and Defect Detections in Food and Agricultural Industry: A Review. J. Agric. Food Res..

[45] Ordoñez-Araque R., Rodríguez-Villacres J., Urresto-Villegas J. (2020). Electronic Nose, Tongue and Eye: Their Usefulness for the Food Industry. Vitae.

[46] Mahmood L., Ghommem M., Bahroun Z. (2023). Smart Gas Sensors: Materials, Technologies, Practical Applications, and Use of Machine Learning—A Review. J. Appl. Comput. Mech..

[47] Anwar H., Anwar T., Murtaza S. (2023). Review on Food Quality Assessment Using Machine Learning and Electronic Nose System. Biosens. Bioelectron. X.

[48] Galvan D., Aquino A., Effting L., Mantovani A.C.G., Bona E., Conte C.A. (2021). E-Sensing and Nanoscale-Sensing Devices Associated with Data Processing Algorithms Applied to Food Quality Control: A Systematic Review. Crit. Rev. Food Sci. Nutr..

[49] Wang A., Zhu Y., Zou L., Zhu H., Cao R., Zhao G. (2022). Combination of Machine Learning and Intelligent Sensors in Real-Time Quality Control of Alcoholic Beverages. Food Sci. Technol..

[50] Rodríguez S.D., Barletta D.A., Wilderjans T.F., Bernik D.L. (2014). Fast and Efficient Food Quality Control Using Electronic Noses: Adulteration Detection Achieved by Unfolded Cluster Analysis Coupled with Time-Window Selection. Food Anal. Methods.

[51] Menon S., Jain K. (2021). Blockchain Technology for Transparency in Agri-Food Supply Chain: Use Cases, Limitations, and Future Directions. IEEE Trans. Eng. Manag..

[52] Song C.Y., Wu Z.P., Gray J., Meng Z.Z. (2024). An RFID-Powered Multisensing Fusion Industrial IoT System for Food Quality Assessment and Sensing. IEEE Trans. Ind. Inf..

[53] Yu B., Zhan P., Lei M., Zhou F., Wang P. (2020). Food Quality Monitoring System Based on Smart Contracts and Evaluation Models. IEEE Access.

[54] Akhtaruzzaman Khan M., Emran Hossain M., Shahaab A., Khan I. (2022). ShrimpChain: A Blockchain-Based Transparent and Traceable Framework to Enhance the Export Potentiality of Bangladeshi Shrimp. Smart Agric. Technol..

[55] Oates M.J., Gonzalez-Teruel J.D., Ruiz-Abellon M.C., Guillamon-Frutos A., Ramos J.A., Torres-Sanchez R. (2022). Using a Low-Cost Components e-Nose for Basic Detection of Different Foodstuffs. IEEE Sens. J..

[56] Karakaya D., Ulucan O., Turkan M. (2020). Electronic Nose and Its Applications: A Survey. Int. J. Autom. Comput..

[57] Coombs C.E.O., Allman B.E., Morton E.J., Gimeno M., Horadagoda N., Tarr G., González L.A. (2022). Differentiation of Livestock Internal Organs Using Visible and Short-Wave Infrared Hyperspectral Imaging Sensors. Sensors.

[58] Lam M.B., Nguyen T.H., Chung W.Y. (2020). Deep Learning-Based Food Quality Estimation Using Radio Frequency-Powered Sensor Mote. IEEE Access.

[59] Siddiqui J., Taheri M., Ul Alam A., Deen M.J. (2022). Nanomaterials in Smart Packaging Applications: A Review. Small.

[60] Huang W.T., Wang X.P., Xia J., Li Y.L., Zhang L.W., Feng H.H., Zhang X.S. (2023). Flexible Sensing Enabled Agri-Food Cold Chain Quality Control: A Review of Mechanism Analysis, Emerging Applications, and System Integration. Trends Food Sci. Technol..

[61] Chung W.Y., Le G.T., Tran T.V., Nguyen N.H. (2017). Novel Proximal Fish Freshness Monitoring Using Batteryless Smart Sensor Tag. Sens. Actuators B-Chem..

[62] Ahari H., Akbari-Adreghani B., Razavilar V., Motallebi A., Moradi S., Anvar A.A. (2014). The Staphylococcus Aureus Exotoxin Recognition Using a Sensor Designed by Nanosilica and SEA Genotyping by Multiplex PCR. Appl. Food Biotechnol..

[63] Deepa S.N., Jayalakshmi N.Y. (2022). An Intelligent Neural Network Algorithm for Uncertainty Handling in Sensor Failure Scenario of Food Quality Assurance Model. Comput. Assist. Methods Eng. Sci..

[64] Kaya A., Keçeli A.S., Catal C., Tekinerdogan B. (2020). Sensor Failure Tolerable Machine Learning-Based Food Quality Prediction Model. Sensors.

[65] Wijaya D.R., Sarno R., Zulaika E. (2019). Noise Filtering Framework for Electronic Nose Signals: An Application for Beef Quality Monitoring. Comput. Electron. Agric..

[66] Barandun G., Gonzalez-Macia L., Lee H.S., Dincer C., Güder F. (2022). Challenges and Opportunities for Printed Electrical Gas Sensors. ACS Sens..

[67] Zhang D., Yu S., Wang X., Huang J., Pan W., Zhang J., Meteku B.E., Zeng J. (2022). UV Illumination-Enhanced Ultrasensitive Ammonia Gas Sensor Based on (001)TiO2/MXene Heterostructure for Food Spoilage Detection. J. Hazard. Mater..

[68] Mohammed M., Riad K., Alqahtani N. (2022). Design of a Smart IoT-Based Control System for Remotely Managing Cold Storage Facilities. Sensors.

[69] Kittichotsatsawat Y., Jangkrajarng V., Tippayawong K.Y. (2021). Enhancing Coffee Supply Chain towards Sustainable Growth with Big Data and Modern Agricultural Technologies. Sustainability.

[70] Zhu G.N., Zeng Y., Teoh Y.S., Toh E., Wong C.Y., Chen I.M. (2023). A Bin-Picking Benchmark for Systematic Evaluation of Robotic-Assisted Food Handling for Line Production. IEEE/ASME Trans. Mechatron..

[71] Yu Z.L., Jung D.Y., Park S., Hu Y.X., Huang K., Rasco B.A., Wang S., Ronholm J., Lu X.N., Chen J.H. (2020). Smart Traceability for Food Safety. Crit. Rev. Food Sci. Nutr..

[72] Chatterjee K., Singh A., Neha (2023). A Blockchain-Enabled Security Framework for Smart Agriculture. Comput. Electr. Eng..

[73] Ehsan I., Khalid M.I., Ricci L., Iqbal J., Alabrah A., Ullah S.S., Alfakih T.M. (2022). A Conceptual Model for Blockchain-Based Agriculture Food Supply Chain System. Sci. Program..

[74] van Hilten M., Ongena G., Ravesteijn P. (2020). Blockchain for Organic Food Traceability: Case Studies on Drivers and Challenges. Front. Blockchain.

[75] Zhang X., Sun P.C., Xu J.P., Wang X.Y., Yu J.B., Zhao Z.Y., Dong Y.F. (2020). Blockchain-Based Safety Management System for the Grain Supply Chain. IEEE Access.

[76] Nurgazina J., Pakdeetrakulwong U., Moser T., Reiner G. (2021). Distributed Ledger Technology Applications in Food Supply Chains: A Review of Challenges and Future Research Directions. Sustainability.

[77] Lin W., Huang X., Fang H., Wang V., Hua Y., Wang J., Yin H., Yi D., Yau L. (2016). Blockchain Technology in Current Agricultural Systems: From Techniques to Applications. IEEE Access.

[78] Jing R., Li P. (2022). Quality Control System of Red Jujube by Hybrid Model: Development of an Efficient Framework. Front. Plant Sci..

[79] Creydt M., Fischer M. (2019). Blockchain and More—Algorithm Driven Food Traceability. Food Control.

[80] Prashar D., Jha N., Jha S., Lee Y., Joshi G.P. (2020). Blockchain-Based Traceability and Visibility for Agricultural Products: A Decentralizedway of Ensuring Food Safety in India. Sustainability.

[81] Pandey V., Pant M., Snasel V. (2022). Blockchain Technology in Food Supply Chains: Review and Bibliometric Analysis. Technol. Soc..

[82] Peng X., Zhang X., Wang X., Li H., Xu J., Zhao Z. (2022). Multi-Chain Collaboration-Based Information Management and Control for the Rice Supply Chain. Agriculture.

[83] Qian J., Yu Q., Jiang L., Yang H., Wu W. (2022). Food Cold Chain Management Improvement: A Conjoint Analysis on COVID-19 and Food Cold Chain Systems. Food Control.

[84] da Silva F.T., Baierle I.C., Correa R.G.d.F., Sellitto M.A., Peres F.A.P., Kipper L.M. (2023). Open Innovation in Agribusiness: Barriers and Challenges in the Transition to Agriculture 4.0. Sustainability.

[85] Rao S., Chen F., Hu W., Gao F., Huang J., Yi H. (2023). Consumers’ Valuations of Tea Traceability and Certification: Evidence from a Blockchain Knowledge Experiment in Six Megacities of China. Food Control.

[86] Heinonen K. (2023). Characterizing Ex Situ Value: A Customer-Dominant Perspective on Value. J. Travel. Res..

[87] Fadilasari D.P., Roy Ghatak R., Garza-Reyes J.A., Joshi R., Kandasamy J. (2024). Adopting Quality Management Practices in the Industry 4.0 Era: An Investigation into the Challenges. Total Qual. Manag. Bus. Excell..

[88] Yfanti S., Sakkas N. (2024). Technology Readiness Levels (TRLs) in the Era of Co-Creation. Appl. Syst. Innov..

[89] Duong L.N.K., Al-Fadhli M., Jagtap S., Bader F., Martindale W., Swainson M., Paoli A. (2020). A Review of Robotics and Autonomous Systems in the Food Industry: From the Supply Chains Perspective. Trends Food Sci. Technol..

[90] Deng W., Alias S.N., Md Rami A.A., Ismail I.A. (2023). Antecedents of Resistance to Organizational Change: A Systematic Literature Review. Int. J. Acad. Res. Bus. Soc. Sci..

[91] Pittri H., Godawatte G.A.G.R., Esangbedo O.P., Antwi-Afari P., Bao Z. (2025). Exploring Barriers to the Adoption of Digital Technologies for Circular Economy Practices in the Construction Industry in Developing Countries: A Case of Ghana. Buildings.

