# Supplementary material for: Advances in Food Quality Management Driven by Industry 4.0: A Systematic Review-Based Framework
Source: Foods. 2025 Jul 10;14(14):2429. doi: 10.3390/foods14142429 (PMC12294287; doi:10.3390/foods14142429)
Supplement: Supplementary file 1 [file foods-14-02429-s001.zip › foods-3672377-supplementary.pdf]

Table S1. Quality design activities supported by digital technologies.

**QD clusters**				**Digital technologies**								
**Process development**	**Product development**	**New material development**	**Examples**	**BC**	**IoT**	**SS**	**AI**	**ML**	**DT**	**BD**	**RB**	**Reference**
X	X		• DT for the development of a new quality process for cooling horticultural products, as well as the proposition of new packaging designs and cooling installation projects.			X			X			[26]
	X	X	• Customization of high-quality products using 3D printing and supported by big data from various food groups.<br>• Big data, AI, and multi-scale modeling techniques for pre-formulation studies and the prediction of nutritional effects.<br>• AI and big data to support decision-making on packaging materials.				X					[9]
	X	X	• Analysis of information on consumer perspectives using deep learning algorithms for new product development.<br>• Identification of optimal packaging design and materials to improve product quality and extend shelf-life.				X					[15]
X	X		• DT and smart sensor allowing monitored real data to be evaluated against designed product specifications and customer requirements.<br>• DT technology for simulations to provide information on reducing project costs, time, and resources without needing prototype testing.<br>• DT can be used to clarify specifications with suppliers and optimize projects by improving cross-functional team collaboration.<br>• Development of a new DT for each product so that the analysis of compliance with quality requirements during product launch will be made easier.			X			X			[27]
**Process development**	**Product development**	**New material development**		**BC**	**IoT**	**SS**	**AI**	**ML**	**DT**	**BD**	**RB**	
**2**	**4**	**2**	**TOTAL**	**0**	**0**	**2**	**2**	**0**	**2**	**0**	**0**	

Table S2. Quality control activities supported by digital technologies.

QC clusters										Digital Technologies								
**Classification**	**Detection**	**Monitoring**	**Inspection**	**Fraud prevention**	**Prediction**	**Assessment**	**New analysis**	**New sensor**	**Examples**	**BC**	**IoT**	**SS**	**AI**	**ML**	**DT**	**BD**	**RB**	**Reference**
X	X			X					• Recognition and analysis of the degree of ripeness and crispness in the industrial processing of dehydrated strawberries.<br>• Classification of the quality of dehydrated carrots.<br>• Analysis of industrial spray-drying of strawberry juice powder.<br>• AI and ML integration with fluorescence sensors, ultrasound, dielectric/laser spectroscopy, or e-noses for quality control and authentication of olive oil.				X					[10]
		X				X			• Remote sensing enables the diagnosis of temperature and the assessment of quality loss in fresh horticultural produce located in areas with limited accessibility for standard point-probe sensors, such as the center of a fruit pallet.			X			X			[26]
	X	X	X			X	X		• AI integration enhances the analytical performance of spectroscopic systems.<br>• AI used in conjunction with simple sound vibrations traversing the food product to verify high-quality products with no additives and organic food products.<br>• AI for monitoring the freshness of meat using biogenic amines biomarkers.<br>• Non-destructive inspection based on X-ray CT scans used with a deep neural network to indicate suboptimal storage conditions of pear fruits and to detect internal disorders, such as internal browning and cavity formation.<br>• AI image recognition enables the estimation of food nutrient content through the analysis of visual data.				X					[9]
		X	X						• Defects, contaminants, and inconsistencies identification in the agri-food sector, improving efficiency, reducing waste, and raising food safety and quality, enabling intelligent decision-making.				X					[15]

							• Product sorting allowing various aspects of food to be analyzed and deficiencies to be identified in real time, without the need to wait until the end of the production cycle to identify problems and resolve them.								
		X			X		• Food manufacturing simulation using DT to provide relevant product and process information, contributing to real-time control, troubleshooting, and supply chain management.		X			X			[27]
		X	X	X			• Sensor fusion enables online, real-time inspection of raw materials and processing conditions.<br>• IoT sensors based on UV–visible–near infrared spectroscopy, in collaboration with big data, for monitoring the quality and authenticity of food composition.	X	X	X			X		[17]
	X	X					• Evolution of intelligent sensor systems for food monitoring based on advances in printed antennas and the proposal of battery-free RFID sensors to enable greater autonomy for the detection, processing, and supply of data by the sensor system.		X						[38]
		X	X				• A step-by-step procedure proposition with guidelines for developing appropriate algorithms prior to the application of AI model in food industry quality control activities.		X	X					[16]
X	X		X	X	X	X	• Computer Vision System to analyze cheese quality attributes and to identify clusters of spoiled grapes incapable of producing wine.<br>• ANN to report fermentation, color, turbidity, foam volume and persistence, clarity, mouthfeel, and bitterness of beer.<br>• E-tongue to check the quality of different types of beverages.<br>• Deep learning to classify apples.<br>• ANN for shelf-life prediction of cheese at 30°C, considering pH, spore, and mold counts as input parameters and sensory analysis as an output parameter.			X				X	[18]
X	X			X	X		• PLSR to distinguish banana, cherry, and peach ripeness and predict the spatial distribution of measured ripeness characteristics.<br>• PLSR and SVM combined with wavelength selection algorithms to detect and visualize mechanical damage in blueberries and apples.<br>• Regression models are employed to predict the concentration of adulterants in almond flour, tapioca flour, whole wheat flour, cumin powder, and minced pork.<br>• ANN combined with nonlinear mapping is used to classify adulteration in chocolate powder, red pepper powder, and cereals, among other applications.<br>• SVM and PLS-DA to identify varieties, origins, and quality of agricultural products such as tea, coffee beans, cocoa beans, and chia seeds.				X				[19]

X	X				X				• ML tools facilitate the detection of mechanical damage and cold injury, the prediction of firmness in fruit, and the identification and differentiation of classes. Furthermore, ML enables the determination of chemical composition and varieties, the detection of contamination levels, and the determination of soluble solids content, among other applications.				X	[20]
		X							• RFID tags integrated into packaging enable the monitoring and control of process conditions for fresh produce via the IoT, facilitating informed decision-making by stakeholders.	X	X			[23]
	X	X		X		X			• Imaging sensors and spectroscopy for detection of bruises in mushrooms, the monitoring of color in meats, the fatty acid composition of milk, the determination of trans fatty acids in edible oils and to assess the adulteration of powdered milk, pork, beef, and shrimp.<br>• E-nose as sensory and off-flavor analysis in catfish fillets and analysis of firmness, sugar content, and acidity in peaches.<br>• Sensor-type "spectroscopy" for the authentication of species and the different muscle groups within chicken, pork, turkey, lamb, and beef species, as well as the detection of adulteration in beef, turkey, and hamburger steak.<br>• SVM for assessment of spoilage in fillet steak.<br>• PCA for detection of microbial contamination in tomatoes.<br>• PCA and ANN for detection of fungal diseases in strawberries.		X			[21]
X		X							• Particle sensors are employed to track the release of total volatile organic compounds from pork and fish packages, and this data is combined with ML tools to classify food freshness.		X			[57]
	X								• Sensor-based detection of pathogens and toxins in food and drinks.		X			[13]
X			X						• PLS-DA and random forest methods applied on the classification of bovine and ovine parenchymal organs (heart, kidney, liver, and lung).		X			[56]
		X							• RFID technologies applied to transmit information collected by gas sensor array in smart packaging.		X			[29]
		X							• Physical and chemical food parameters monitoring using chipless RFID sensors.		X			[31]
	X	X				X		X	• TC-PDI/CdS micro belt sensor for ultrasensitive detection of trimethylamine, a toxic gas released during the deterioration of marine fish.		X			[39]
						X		X	• Battery-free smart tag sensor proposal to predict the quality of packaged fish by measuring the temperature and concentration of hydrogen sulfide and ammonia gases.		X			[60]

								X	• Nanomaterials are employed in pH indicators and time–temperature indicators to enhance smart packaging applications.	X			[58]
								X	• The utilization of flexible sensing technology enables the optimization of packaging performance, the extension of the shelf-life of frozen products, and the determination of the opening state of packages.	X			[59]
		X	X					X	• An automated framework that uses a network of sensors to measure temperature, humidity, and CO_2 or CH_4 and compares the results with previously established specifications of fruit and vegetable processing industry.	X			[33]
		X						X	• Middleware architecture incorporating a decision support system is employed to transmit information on vital parameters—such as temperature, aroma, and the presence of microorganisms—to process supervisors in the event of quality problems within industrial plants located in remote areas.	X			[34]
	X				X				• AI-based methods for detection and prediction during the processes of selecting, drying, disinfecting, sterilizing, and freezing berries.		X		[42]
	X	X		X	X				• AI is integrated into the sensors to detect errors during the manufacturing process and rectify problems.<br>• E-noses are employed to predict shelf-life and identify food adulteration.<br>• A light scattering sensor for the detection of the three major food pathogens in food.<br>• A biosensor synthesized for the detection of allergenic milk proteins.	X	X		[14]
X	X			X		X			• PCA is integrated with sensors to evaluate meat degradation and detect toxins in packaged products.<br>• PCA and SVM for the detection of thermal degradation of vegetable oil.<br>• Discrete wavelets transform long short-term memory model to classify beef quality and estimate total viable count.<br>• E-nose and ML for the detection of contaminants in vegetable oils such as sunflower and olive oil, rice grains, and saffron.<br>• E-nose fuzzy controller system to identify the presence of *Sitophilus oryzae* infestation in rice grain.			X	[45]
X	X	X							• E-noses are applied in the detection of flaws during the fermentation of alcoholic beverages.<br>• E-tongues are employed for monitoring of variation in alcohol content of beer during fermentation, controlling wine fermentation, and discriminating wine samples during production.	X			[48]
X						X			• E-noses are employed for the determination of the degree of roasting of cocoa beans, the distinction between flavors of tea samples, and the quality assessment of different origins of instant coffees.	X			[55]

X	X	X		X	X	X			• E-noses are employed for the detection of defects and classification of hops, barley, and yeasts and types of varieties in the manufacture of beers, the discrimination between volatile aromatic compounds of different flours and ingredients used in the formulation, processing and storage conditions of bakery products, for the differentiation between different types of spices, for the detection and differentiation of mycotoxigenic fungal strains in contaminated grains, for the assessment of semi-quantitative/qualitative mycotoxin contamination, for the early detection of spoilage and fungal growth in cereals, and for the identification of spoilage in bread.<br>• E-eyes are applied for monitoring product aging, detecting foreign substances, checking for color change during cooking, frying, and freezing of food, and predicting ripeness parameters of citrus fruits.<br>• Sensors for the milk adulteration analysis.		X	[44]
	X				X	X			• E-tongues are applied for the evaluation of the pungency of capsaicin in spicy foods.<br>• E-sensing is applied to predict heavy metals and to detect food-borne bacterial pathogens.		X	[47]
						X		X	• Biosensor with nanotechnology is employed to diagnose *Staphylococcus aureus* with reduced response time, allowing products to be released more quickly.		X	[61]
		X				X			• A monitoring framework combines quality assessment smart contracts and models for automatic quality assessment of fruit juice samples collected from different production stages.	X		[52]
		X				X			• E-noses are applied to monitor the growth of spoilage bacteria in milk and for the quality assessment of modified atmosphere packaged chicken meat.		X	[32]
	X			X					• E-noses are employed to detect the adulteration of commodity foods such as meat, oil, honey, dairy products, tea, and coffee.		X	[46]
X	X		X	X			X		• New unfolded cluster analysis to selected windows method is employed to quickly detect subtle differences in aromatic profiles by analyzing only a few samples of green coffee beans and Cayenne.		X	[49]
	X		X	X			X		• A small sensors system incorporating nanometric chemical sensors analyzes volatile compounds and rapidly determines the authenticity of Parmigiano Reggiano, as well as identifying food pathogens and microbiological contamination, such as *Campylobacter jejuni*.		X	[41]
X	X			X			X		• An e-nose based on an 8 MQ gas sensor array and integrated with ML algorithms is employed to discriminate between samples of different types of meat and fish.		X	[54]

X	X					X	X	• Microbiological verification based on organic field-effect transistors, when combined with ML tools, enables the separation of foods into different types, such as pork, chicken, fish, or milk. It also detects gases released during the decomposition of these foods at concentrations two orders of magnitude below the established safe consumption threshold.			X		[40]
				X	X		X	• A multi-sensor fusion system, applying ML tools, predicts equivalent total volatile organic compounds to ensure the future quality of ham products and mitigate the risk of adulteration.			X		[51]
X	X			X				• A guide is available for the selection of AI techniques for the prediction of food quality authentication and classification of agricultural products.				X	[43]
		X		X				• Blockchain application in food traceability promotes enhanced monitoring of materials and production processes for product packaging, improved cold chain management, ensured traceability in complex global supply chains, and efficient containment of food fraud.	X				[78]
				X				• A sensor system is used to record every aspect of the craft beer-making process, with blockchain technology employed for secure data storage, thereby ensuring the transparency and authenticity of ingredients and the brewing process.	X				[50]
		X						• A food supply chain management system, leveraging sensors and blockchain technology, informs authorized users about the condition of monitored food. Upon the detection of abnormal conditions, the system alerts the manager, who then directs the process controller to take necessary action.			X		[36]
		X				X		• A blockchain-based wireless sensor network monitoring system collects environmental parameters (temperature, humidity, gas, etc.) during the cold storage of frozen seafood, enabling prediction and quality assessment through the application of ML tools. This system facilitates improved management of critical control points, ensures product quality and safety, and mitigates quality loss by enhancing the control of freezing environment parameters.			X		[35]
				X		X		• Blockchain technology enables the identification of lower quality shrimp products that have been mislabeled as premium within the shrimp supply chain.	X				[53]

QC clusters										Digital Technologies							
**Classification**	**Detection**	**Monitoring**	**Inspection**	**Fraud prevention**	**Prediction**	**Assessment**	**New analysis**	**New sensor**		**BC**	**IoT**	**SS**	**AI**	**ML**	**DT**	**BD**	**RB**
**14**	**21**	**23**	**9**	**17**	**9**	**16**	**6**	**7**	**TOTAL**	**4**	**2**	**33**	**9**	**3**	**2**	**1**	**1**

Table S3. Quality improvement activities supported by digital technologies.

QI clusters					Digital technologies								
**Process variability reduction**	**Waste reduction**	**Continuous improvement**	**Performance enhancement sensor**	**Examples**	**BC**	**IoT**	**SS**	**AI**	**ML**	**DT**	**BD**	**RB**	**Reference**
X				• AI combined with big data to optimize the batch blending process and predict product quality.<br>• AI and big data to adjust the ambient temperature of fresh food stock more quickly than in traditional systems and keep the food consistently at the optimum storage temperature, increasing its quality.				X					[9]
	X	X		• DT to visualize the evolution of the process without the need to stop equipment or open the system to examine its state physically.<br>• Early detection of system failures by DT contributes to a more efficient approach to predictive maintenance and optimizes the uniformity, performance, and sustainability of processes.			X			X			[27]
X		X		• Proposal for a smart IoT-based control system that connects multipurpose sensors, actuators, and cold storage-related equipment via IoT and provides reliable data on the indoor microclimate atmosphere, the applied electric current, and the energy consumption of a modified cold storage room and sends the necessary alerts in case of emergency based on real-time data analysis of fruits' post-harvest quality.		X	X						[67]
		X		• BD from the coffee manufacturing process (temperature, pH, and humidity from the washing, fermentation, drying, and storage stages) being analyzed by integrated systems aiming to eliminate the frequency of human monitoring, mitigating the possibility of error, helping to reduce costs and time, and increasing the effectiveness and efficiency of the coffee supply chain.							X		[68]
X		X		• A framework proposition for raw and cooked food fractionation in an in-flight catering center or lunch box factory using robots in the food handling process, which aims to raise the quality level of the products by working with smaller process tolerances.								X	[69]
			X	• A framework proposition that filters out noise-contaminated e-nose signals for beef quality classification, minimizing sensor drift.					X				[64]

			X	• Technique proposition based on the Single Plurality Voting System (SPVS) classification approach to tolerate and ignore sensor failures, minimizing sensor drift, and automatically continuing the quality assessment and keeping the prediction accuracy of the beef cut quality assessment model acceptable.					X				[63]
			X	• Proposition of a food quality monitoring model based on AI olfaction that fills in missing data during collection, minimizing sensor drift and presenting efficient classification performance (in situations of sensor failure) applied to wine and meat quality verification database.			X						[62]
			X	• Printed electrical gas sensors used to improve the performance of ammonia sensors in detecting spoilage of protein-rich products and fruit ripening, providing these sensors with high-performance, low-cost, and low-energy consumption.			X						[65]
			X	• Development of a high-performance gas sensor to detect traces of ammonia in spoiled pork, fish, and shrimp in real time.			X						[66]
**QI clusters**					**Digital technologies**								
**Process variability reduction**	**Waste reduction**	**Continuous improvement**	**Performance enhancement sensor**		**BC**	**IoT**	**SS**	**AI**	**ML**	**DT**	**BD**	**RB**	
**3**	**1**	**4**	**5**	**TOTAL**	**0**	**1**	**5**	**1**	**2**	**1**	**1**	**1**	

Table S4. Quality assurance activities supported by digital technologies.

QA clusters									Digital technologies								
**Quality management programs**	**Quality check**	**Quality system**	**Traceability**	**Contract compliance**	**Quality Audit**	**Safety hazards**	**Certification**	**Examples**	**BC**	**IoT**	**SS**	**AI**	**ML**	**DT**	**BD**	**RB**	**Reference**
X								• Combination of sensors with DT has been reported to support HACCP in horticultural products, since DT technology can assist feedback control, establish corrective actions, and perform predictive model control.			X			X			[26]
X	X	X						• Image analysis can be employed to detect foreign objects in food, as exemplified by a cylindrical tactile image sensor reported to detect residual shrimp shell fragments on the crustacean's surface and bones within fish fillets.<br>• Ultrasonic sensing imaging and optical fluorescence methods are employed to determine the requisite level of cleaning on equipment, as exemplified by the Self-Optimizing Clean-In-Place system, which autonomously optimizes the cleaning process of food manufacturing equipment.				X					[15]
	X	X						• AI can be implemented to ensure, through facial and object recognition, that employees wear appropriate personal protective equipment, such as masks or caps, and that they verify temperatures and the cleanliness of food.				X					[14]
			X					• Smart sensors provide real-time information for customers about relevant changes in product specification, changes in the product and environment, product history, and authenticity improving product traceability.			X						[70]
	X		X	X	X			• A multi-agent system (producer, manufacturing, transportation, and retail) with smart contracts to track the origin of agricultural food, quality check of products, and monitoring of products shipment.<br>• Agents can also check that both parties are following the terms and conditions of the contract and fines or penalties can be applied.	X								[72]

						• Blockchain to support much easier and faster auditing without involving so many people.		
X	X		X			• A blockchain-based Xinzheng red jujube quality traceability system aids in minimizing the production and distribution of unsafe or low-quality products, thereby reducing the potential for negative publicity, liability, and recalls. Among the system's modules, data auditing relies on quality test data stored on the blockchain to audit the quality inspection function.	X	[77]
	X					• A hybrid public–private blockchain, termed ShrimpChain, feeds smart contracts with relevant data from all production stages, enabling a distributed and cumulative certification approach based on a score that ranks packaged shrimp according to the integrity and accuracy of the authenticated data.	X	[53]
	X					• A blockchain-based food supply chain system utilizes smart contracts and issues a certificate for each food item verified during purchase. Key stakeholders are interconnected via the blockchain, and IoT devices attached to the products facilitate automated data collection and upload to the blockchain network, eliminating human intervention.	X	[71]
X	X					• A prototype system for information supervision of the rice supply chain based on a multi-chain collaboration architecture that integrates blockchain, IoT, and cloud technologies for real-time information management and control.	X	[81]
X	X					• A prototype frozen food chain management system that monitors real-time parameters of the cold chain environment (temperature, humidity, gas such as ethylene, position, and lighting) and a blockchain platform, which uses smart contracts to establish incident alert levels. Thus, in a food cold chain failure scenario for perishable products, intelligent decision-making can be carried out automatically.	X	[82]
	X			X		• To manage business data and hazard information, enhance data reliability, and mitigate risk, a customized smart contract was employed to control data reading and writing. This smart contract is defined in accordance with relevant laws, regulations, and standards, thereby assisting companies and regulatory authorities in promptly investigating and addressing potential safety hazards and preventing quality and safety incidents.	X	[74]
	X				X	• Blockchain technology facilitates a more precise determination of the origin of organic and fair-trade food compared to annual sampling within a third-party certification process, as demonstrated in case studies involving nutmeg, tuna, citrus fruits, and rice.	X	[73]

	X	• A blockchain-based supply chain possesses the potential to enable closer collaboration among quality control agents, food administration agencies, producers, and manufacturers, thereby simplifying the traditionally protracted quality monitoring and auditing process.	X		[76]
X	X	• Implementation of a blockchain-based IoT food traceability system enhances information sharing throughout the food chain, as traceability information—encompassing processing conditions—is released by the manufacturing company. Transparent data management provides a reliable means for numerous operational activities, such as audit checking.	X	X	[24]
X	X	• For food traceability, a blockchain-based secure and decentralized food industry architecture connects producers, manufacturers, and food regulators with government agencies and the general population, providing a secure and safe food system against security threats. This security is enhanced using IoT sensors that send data to smart contracts and validate users.<br>• The auditable and transparent features inherent in blockchain technology facilitate both the detection of fraud and the mitigation of violations of codes of conduct throughout the food industry. Furthermore, it enables instantaneous traceability of food products through the audit trail.	X	X	[25]
X		• The integration of blockchain and IoT technologies can contribute to the acquisition and dissemination of reliable, real-time product-related information across the supply chain, thereby supporting shelf-life management and facilitating product recalls through data-driven decision-making.	X	X	[75]
X		• A blockchain-based traceability system enables consumers to readily access information concerning the extra virgin olive oil being purchased through smartphone connectivity to the system, which can also be integrated with the databases of public control authorities.	X	X	[10]
X	X	• Enhanced agility in mitigating food crises through the implementation of blockchain-based food traceability, which facilitates the precise detection and elimination of contamination sources.<br>• Objective analytical methods or regular audits of production sites serve as mechanisms for verifying real-world input data within a digitized blockchain, particularly at the initial stages of the process chain. These approaches could conceivably be integrated into the structures of a HACCP system, thereby facilitating food supply chain traceability.	X		[78]

	X		X					• Development and implementation of a blockchain-based solution that enables real-time monitoring and management of all communications and transactions in the agricultural supply chain, aiming to ensure food safety in a decentralized manner.	X								[79]
					X			• Blockchains technology confers value upon consumers, as they are enabled to audit resources and product flows within the system with enhanced knowledge and transparency.	X								[50]
			X		X			• A traceability system for e-commerce agricultural products based on blockchain to ensure these items' food safety and quality.<br>• A blockchain-based information system effectively tracks, monitors, and audits the entire food process, including critical production stages, thereby enabling manufacturers and customers to access comprehensive information regarding the production process.<br>• A blockchain-based solution for the Italian Carasau bread supply chain enables each actor to demonstrate product superiority throughout the chain and assure customers regarding product suitability for consumption.	X	X							[80]
**QA clusters**									**Digital technologies**								
**Quality management programs**	**Quality check**	**Quality system**	**Traceability**	**Contract compliance**	**Quality Audit**	**Safety hazards**	**Certification**		**BC**	**IoT**	**SS**	**AI**	**ML**	**DT**	**BD**	**RB**	
**2**	**4**	**5**	**16**	**1**	**8**	**1**	**1**	**TOTAL**	**17**	**5**	**2**	**2**	**0**	**1**	**0**	**0**	

Table S5. Quality policy and strategy activities supported by digital technologies.

QPS clusters					Digital technologies								
**Strategic Analysis**	**Quality cost**	**Customer focus**	**Quality policy**	**Examples**	**BC**	**IoT**	**SS**	**AI**	**ML**	**DT**	**BD**	**RB**	**Reference**
X	X	X		• Data collected from interviews with tea consumers regarding blockchain-based traceability was utilized to inform the strategic management of investments in food quality and safety programs.	X								[84]
		X		• The integration of blockchain and IoT technologies enhances product traceability, enabling consumers to scrutinize comprehensive product information and verify authenticity via smart contract implementation, as exemplified by the mitigation of fruit fraud risks.	X	X							[10]
		X		• Blockchain-based IoT applications furnish reliable and detailed product information encompassing food origin, logistics, production, and distribution, thereby empowering informed and responsible consumer purchasing decisions.	X	X							[75]
X				• A balanced strategic integration of big data, AI, and blockchain technologies is crucial for end-to-end food quality and safety monitoring, alongside enhanced quality management and product traceability throughout production, distribution, and consumption stages.				X					[9]
X				• Blockchain offers enhanced transparency regarding governmental tax collection and facilitates a more assertive allocation of resources to support producers in meeting domestic and international market demands, thereby promoting the export potential of the nation's shrimp industry.	X								[53]
			X	• Blockchain-derived retailer and consumer data enhances the operational prioritization of governmental and regulatory bodies. Access to regional demand data enables policymakers to more efficiently prioritize and coordinate with all relevant stakeholders in response to unforeseen events.	X								[76]
	X	X		• ML has contributed to the reduction in sensory evaluation costs, the optimization of decision-making processes and the enhancement of business strategies to better address user requirements.			X	X					[16]

X		X		• Big data utilization proves advantageous for formulating and guiding an organization's strategic direction. Comprehensive data integration across all organizational nodes facilitates enhanced efficiency and effectiveness through the optimization of costs, time, and resources, alongside improved product quality. Furthermore, it enables the creation of added value for agricultural products, thereby fostering reliability and customer satisfaction.							X		[68]
X				• A blockchain-based wireless sensor network monitoring system has the potential to enhance quality control decision-making strategies. This can be achieved through mechanisms such as First-Expired-First-Out (FEFO) inventory management, dynamic expiry date implementation, and dynamic pricing systems, all while fostering increased transparency and trust and preventing data tampering.			X						[35]
**QPS clusters**					**Digital technologies**								
**Strategic**	**Quality cost**	**Customer focus**	**Quality policy**		**BC**	**IoT**	**SS**	**AI**	**ML**	**DT**	**BD**	**RB**	
**4**	**2**	**5**	**1**	**TOTAL**	**5**	**2**	**2**	**2**	**0**	**0**	**1**	**0**	
